# Supplementary material for: Insecticide-treated house screening protects against Zika-infected Aedes aegypti in Merida, Mexico
Source: PLoS Negl Trop Dis. 2021 Jan 19;15(1):e0009005. doi: 10.1371/journal.pntd.0009005 (PMC7853519; doi:10.1371/journal.pntd.0009005)
Supplement: Attachment — Submitted filename: PNTD-D-20-00660 Itemized list of specific responses Final.docx [file pntd.0009005.s002.docx]

**Itemized list of specific responses to reviewers' comments**

**PLOS Neglected Tropical Diseases submission (PNTD-D-20-00660)**

Dear

Pattamaporn Kittayapong, Ph.D.

Associate Editor

PLOS Neglected Tropical Diseases

Ann Powers, Ph.D.

Deputy Editor

PLOS Neglected Tropical Diseases

Please find attached the revised version of the manuscript ID PNTD-D-20-00660 titled “*Insecticide-treated house screening protects against Zika-infected Aedes aegypti in Merida, Mexico*” with the modifications kindly suggested by the reviewers.

All the suggestions were followed. Here is the point-by-point response to the valuable observations of the reviewers. All suggested changes are also included in the main document as indicated below:

| **Reviewer Comments** | **Response** |
| --- | --- |
| **Reviewer #1** |  |
| **Summary and General Comments** |  |
| Reviewer #1: The manuscript shows convincing evidence of an impact of Household screening on Aedes aegypti population parameters and Aedes infected mosquitoes. This is should be of high relevance, this type of data on an infection proxy is rare so this should be of high interest to PLOS NTD and the ATD vector control community. | **Many thanks** |
| General Comments  - The paper is clear and well done and makes a convincing argument for the value of HS. | **Many thanks** |
| - Study is of good size but not huge. Took advantage of the high transmission period associated with ZIKV, but there is no discussion of human transmission in the study area or houses.  I understand the study may have not been able to test the population, but the lack of discussion of what sort of disease was seen feels a little like a read flag. ZIKV cases are talked about in the intro but very generally. | **Done. See page 15 of the revised manuscript including track changes. Also, we have evidence from other studies, conducted in Merida, about ZIKV transmission during the period of study. Such information is not discussed in more detail.** |
| -There is not mention of insecticide resistance testing, it would have been important to include if done, and any context would have been useful. My understanding is pyrethroid resistance is high thorough out Mexico, so this issue at least deserves some discussion. | **Done. See page 15-16 of the revised manuscript where we now expand on our extensive evidence of resistance in *Ae. aegypti* from Merida.** |
| -A paragraph in the discussion about the potential limitations of the study would be useful. | **Done See page 18.** |
| -A paragraph in the discussion describing some of the previous work done on Insecticide treated curtains also be important. | **Done. See page 16** |
| -You might mention the importance of this intervention in light of the COVID-19 outbreak. | **Done. See page 18** |
| **Methods** |  |
| -The objectives of the study are clearly articulated and an important hypothesis was stated and tested. | **Many thanks** |
| -Study design: The study design was appropriate, but the total number of Aedes collected was relatively small.  I find the differences convincing, but the represent relatively modest, albeit statistically significant differences. Again not many studies have demonstrated these kind of differences, linked to a proxy for transmission, but I might temper the enthusiasm a little. | **We edited the document and “tempered the enthusiasm” while keeping in perspective the relevance of results. Keep in mind we screened over 500 houses, which it is a large undertaking.** |
| I would also commend the research team in taking advantage the introduction of ZIKV.  Was the study planned prior to ZIKV introduction or were you able to react that quickly.  If not I might describe how you got this trial up and running so quickly to take advantage of measuring ZIKV transmission the value this type of trial can contribute to evidence-base for vector-borne diseases. | **Many thanks.**  **The study was planned and implemented prior to ZIKV introduction. The authors of the current study were working already in the area where the introduction of ZIKV occurred and took advantage of the opportunity to compare the impact before and after the introduction of ZIKV.**  **Originally, the primary goal was to evaluate de entomological efficacy (presence and abundance of the vector). Infection of mosquitoes with arboviruses was already planned but was originally a secondary goal since we cannot predict when and where an outbreak can occur.**  **This illustrates the importance of adult collections (and adult derived indicators including infection) for the evaluation of control methods targeting that stage in comparison with the use of larval indicators.**  **It is now explained in the document (Methods and Discussion)** |
| -No sample size calculation is included - this does not bother me, but trialists would be interested in if the study was powered for a particular effect size. | **Done, see page 7. We now add in more detail our procedures for sample size calculation. Keep in mind we powered the study to detect a difference in indoor Ae. aegypti density, not arbovirus infection.** |
| Reviewer #1: -The article is simple and straight forward and the differences are apparent. -Tables are good. - Any insecticide resistance testing? | **Done, see preceding points** |
| **Results** |  |
| -Statistics look appropriate to me, and Table 1 provides the "raw" numbers, again some care needs to be taken in talking about the significance. | **Noted. We have corrected P values in table.** |
| -ZIKV testing - the authors note the "surprising number of positive pools for ZIKV.  The lack of DENV/CHIKV provides some confidence, but my guess is the more laboratory inclined reviewers might be a bit nervous about this.  You might provide a bit more discussion on this issue.  If we could see the proportion of houses with Aedes aegypti that tested positive for ZIKV it would provide some additional perspective.  Was the laboratory blinded to the cluster allocation? | **Many thanks for the observation.**  **We eliminated "surprising”**  **Dengue and Chikungunya did not circulate in Merida with the same intensity as ZIKV. As reported in other areas of the Americas, ZIKV invasion led to a ‘silent’ period of DENV reporting and likely transmission. We do not believe knowledgeable reviewers will see the dominance of ZIKV and lack of DENV infection as a surprise.**  **The proportion of houses with *Aedes aegypti* that tested positive for ZIKV is in the table.**  **Yes, the laboratory diagnostics was blinded to the cluster allocation. It is now explained in the document (Methods).** |
| - Any insecticide resistance testing? | **No as part of this study, but information on this subject is now included in the document, See page 15-16** |
| **Conclusions** |  |
| -The conclusions are supported by the data presented. | Many thanks |
| -Limitations:  I did not see this addressed directly and would have liked to see some discussion.  Although the results speak for themselves, I feel the conclusions could be tempered a bit.  Again, this is incredibly promising, but I would tone the language down in the abstract at least. | **As described before, we edited the document and “tempered the enthusiasm”, including in the abstract.** |
| Excellent discussion of public health relevance, but I would encourage the authors to mention COVID-19 which makes this type of intervention even more relevant.. | **Now included in the discussion** |
| One thing missing from the discussion, is a review (not necessary to be complete) of other studies using Insecticide treated materials, I believe there are some other studies from the Yucatan by another group and an RCT with less promising results using epi endpoints was recently published by Lenhart et al. in PLOS NTD.  Again I think just tempers the conclusions, may not work everywhere in the same way | **Now included in the discussion** |
| **Reviewer #2** |  |
| **Methods** |  |
| Sample size calculation is not reported. | **We now report sample size. Keep in mind we have powered the study for indoor Ae. aegypti density, not infection.** |
| **Results** |  |
| The quality of images is poor. | **Now improved in the resubmission** |
| Reviewer #2: I strongly invite authors to add the numbers of mosquitoes and houses positive for Zika infected mosquitoes in Table 2 (it is just an additional column). | **As described in the methods, we tested pools of mosquitoes and not individual mosquitoes.** |
| **Conclusions** |  |
| Conclusions are supported by results. | **Many thanks** |
| It is not reported if the sample size calculation has been performed so we do not know if this has affected the power of the analysis. | **We report ample power to detect a difference of the primary endpoint (Ae. aegypti abundance). Now, the lack of data for Ae. aegypti infection due to the invasive nature of ZIKV prevented us to calculate power. However, we believe our strong effect is a convincing evidence of the impact of ITS.** |

On Behalf of all the authors

Prof. Pablo Manrique-Saide

Principal Investigator

UCBE-UADY

E-mail: pablo_manrique2000@hotmail.com
